# High-Resolution Body Surface Potential Mapping in Exercise Assessment of Ischemic Heart Disease

**DOI:** 10.1007/s10439-019-02231-2

**Published:** 2019-02-21

**Authors:** Michał Kania, Roman Maniewski, Rajmund Zaczek, Małgorzata Kobylecka, Anna Zbieć, Leszek Królicki, Grzegorz Opolski

**Affiliations:** 1 Nalecz Institute of Biocybernetics and Biomedical Engineering, Polish Academy of Sciences, Trojdena 4, 02-109 Warsaw, Poland; 2 I Chair and Department of Cardiology, Medical University of Warsaw, Warsaw, Poland; 3 Department of Nuclear Medicine, Medical University of Warsaw, Warsaw, Poland

**Keywords:** High-resolution ECG, Coronary angiography, Single photon emission computed tomography, Myocardial ischemia, Exercise test

## Abstract

Standard 12-lead ECG exercise testing is commonly used for screening of ischemic heart disease (IHD). We studied if high-resolution body surface potential mapping (HR-BSPM) during exercise offers advantages over current standards in noninvasive evaluation of IHD. This study was carried out on 90 IHD patients and 33 healthy controls. The 67-lead HR-BSPM was recorded at rest and during exercise. Twenty-one ECG parameters including classical ST criteria were compared. The effectiveness of methods was verified based on the results of SPECT and coronary angiography. The most effective parameters in the diagnosis of IHD were: amplitude parameter ΔST60 and δT parameter showing T-wave morphology changes during exercise. The sensitivities/specificities of ΔST60 and *δT* parameters for the HR-BSPM were 70/69 and 59/62%, while for the standard 12-lead ECG system they were: 63/62 and 59/56%. These results demonstrate the usefulness of HR-BSPM measurements during exercise. HR-BSPM resulted in higher sensitivities and specificities compared to the standard 12-lead exercise test. The advantage was partially associated with observed ischemic changes outside standard precordial leads position that were not visible when using the standard 12-lead exercise test. This justifies research into the optimization of the number and position of ECG leads in exercise testing.

## Introduction

Ischemic heart disease (IHD) is a leading cause of death, responsible for 15.2 millions of deaths globally in 2016.^35^ The most diagnostically important factor in IHD evaluation is invasive examination of coronary arteries, i.e. coronary angiography (CA). High expectations concerning myocardial viability assessment in IHD are associated with the development of multi-slice computed tomography (CT) and single photon emission computed tomography (SPECT). These techniques are highly effective in IHD diagnostic, but their impact is still limited by cost and availability. Therefore, standard ECG exercise test is used as a screening tool for IHD, since it is helpful to identify high-risk individuals who need to be qualified for revascularization treatment. Although ECG exercise test is non-invasive and a low-cost tool for IHD assessment, its clinical usefulness is limited. Reported values of sensitivity (Se) and specificity (Sp) in detection of coronary artery disease (CAD) are low and significantly varied in the literature. In a meta-analysis study performed by Gianrossi *et al*.^8^ on 147 published reports, mean values of Se and Sp were 68% (SD 16%) and 77% (SD 17%), respectively. This could be due to having an insufficient number and locations of ECG electrodes over the torso, as well as the evaluation relying only on standard ST segment depression criteria,^19^ omitting the remaining part of the ECG signal e.g. exercise-induced changes in QRS or T wave.

High-resolution body surface potential mapping (HR-BSPM) is a new BSPM technique that allows for recording and analysis of a complete distribution of action potentials on the thoracic surface with high spatial, temporal and amplitude resolution. The justification for the use of BSPM in the diagnosis of heart disease has been confirmed in numerous studies.^21^,^32^,^33^ Nowadays active ECG electrodes shielding, the use of low-noise amplifiers and the increase of computational power allows for continuous recording and analysis of good quality data from a large number of ECG leads.^11^ Advantages of ECG signal analysis from a larger number of ECG leads over the standard ECG has been demonstrated in previous studies.^1^,^3^,^21^,^33^ However, most of these studies were devoted to the analysis of ECG maps recorded at rest. The majority of these studies were performed with low resolution data.

So far there is a lack of studies on the use of HR-BSPM in exercise evaluation of IHD. There are a few publications on the use of ECG maps recorded with low resolution during exercise test.^9^,^16^,^26^,^28^ Michaelides *et al*.^24^ demonstrated that increasing the number of analyzed ECG leads during exercise test contributes to improvement in detection of one-vessel CAD. This suggests that HR-BSPM may reveal hidden information important in reliable IHD diagnostics. This is in accordance with our preliminary findings in which ischemic changes in the ECG signal were observed in non-standard electrode locations, often in the absence of changes in standard ECG leads.^13^,^15^–^17^,^34^

The aim of the present study was to investigate whether high-resolution body surface potential mapping enables for diagnosis of IHD with higher Se and Sp than standard 12-lead ECG exercise testing. In particular, the diagnostic efficiencies of classical ST segment depression criteria, as well as of new proposed amplitude-time parameters were investigated.

## Materials and Methods

### Study Population

This study was carried out on 90 patients (age 61 ± 9 years) and 33 healthy controls (HC) (age 55 ± 15 years). Subjects were recruited among clinically stable patients referred for myocardial perfusion SPECT imaging at the Department of Nuclear Medicine, Medical University of Warsaw, Poland. These were patients with known coronary disease or with significant clinical suspicion of CAD, risk factors for CAD and chest pain for diagnosis, patients with electrocardiographically positive stress test qualified for coronarography, patients after myocardial infarction treated with coronary angioplasty with the presence of other border stenoses in the coronary arteries requiring an estimation of significance before the decision on further revascularization.

HC had no history of cardiovascular disease, had normal resting 12-lead ECG and no signs of ischemia in 12-lead ECG exercise stress testing.

Exclusion criteria for the study included known contraindications to the exercise test, such as recently myocardial infarction, symptoms of unstable coronary heart disease, significant reduction or elevation of ST in resting ECG not found in previous records, apparent cardiac failure, arrhythmias worsening over time effort, aortic valve stenosis, recent thrombophlebitis or recent arterial embolism, acute infectious diseases, aortic dissecting aneurysm, severe organ and systemic diseases, severe hypertension (> 200/110), intraventricular conduction abnormalities or left ventricular hypertrophy that may affect the correct interpretation of the exercise ECG.

The study was approved by the Bioethics Committee of the Medical University of Warsaw in accordance with the Declaration of Helsinki and informed consent was obtained from each patient.

Studied groups were subdivided based on the outcomes of myocardial perfusion SPECT imaging and CA. Clinical characteristics of studied groups are presented in Table 1.

**Table 1:** Characteristics of the study population.

	HC	grSPECT−	grSPECT+	CA−	CA+	grSPECT−CA−	grSPECT+CA+
Group size (*n*)	33	42	48	42	43	16	27
Age (years)	55 ± 15	62 ± 9	61 ± 9	61 ± 10	61 ± 10	62 ± 7	61 ± 9
Height (cm)	178 ± 7	173 ± 7	173 ± 5	174 ± 6	173 ± 6	176 ± 7	173 ± 5
Weight (kg)	84 ± 14	85 ± 18	81 ± 11	87 ± 16	79 ± 12	87 ± 22	78 ± 12
BMI (kg/m^2^)	26 ± 3	28 ± 6	27 ± 4	29 ± 5	26 ± 4	28 ± 6	26 ± 4
HR at rest/at stress (bpm)	75 ± 16/144 ± 20	64 ± 10/122 ± 15	65 ± 14/118 ± 15	63 ± 10/112 ± 19	65 ± 15/115 ± 19	63 ± 10/122 ± 10	66 ± 16/119 ± 14
LVEF (%)	n/a	55 ± 10	49 ± 10	54 ± 11	46 ± 10	54 ± 13	45 ± 10
MI/ischemia (*n*) Location of MI/ischemia (n):	n/a	27/0	27/48	27/0	19/43	12/0	18/27
Anterior	n/a	6/0	4/14	4/2	4/10	3/0	3/10
Lateral	n/a	6/0	6/13	2/1	8/8	2/0	5/8
Inferior	n/a	23/0	24/35	14/7	25/19	10/0	16/19
Posterior	n/a	17/0	10/18	9/4	13/9	8/0	8/9
Apex	n/a	6/0	4/7	3/0	3/5	3/0	2/5

Data are presented as mean ± SD or by number of subjects (n)

*MI* myocardial infarction, *n/a* not available, *BMI* body mass index, *LVEF* left ventricular ejection fraction

### Data Acquisition

**Figure 1:**
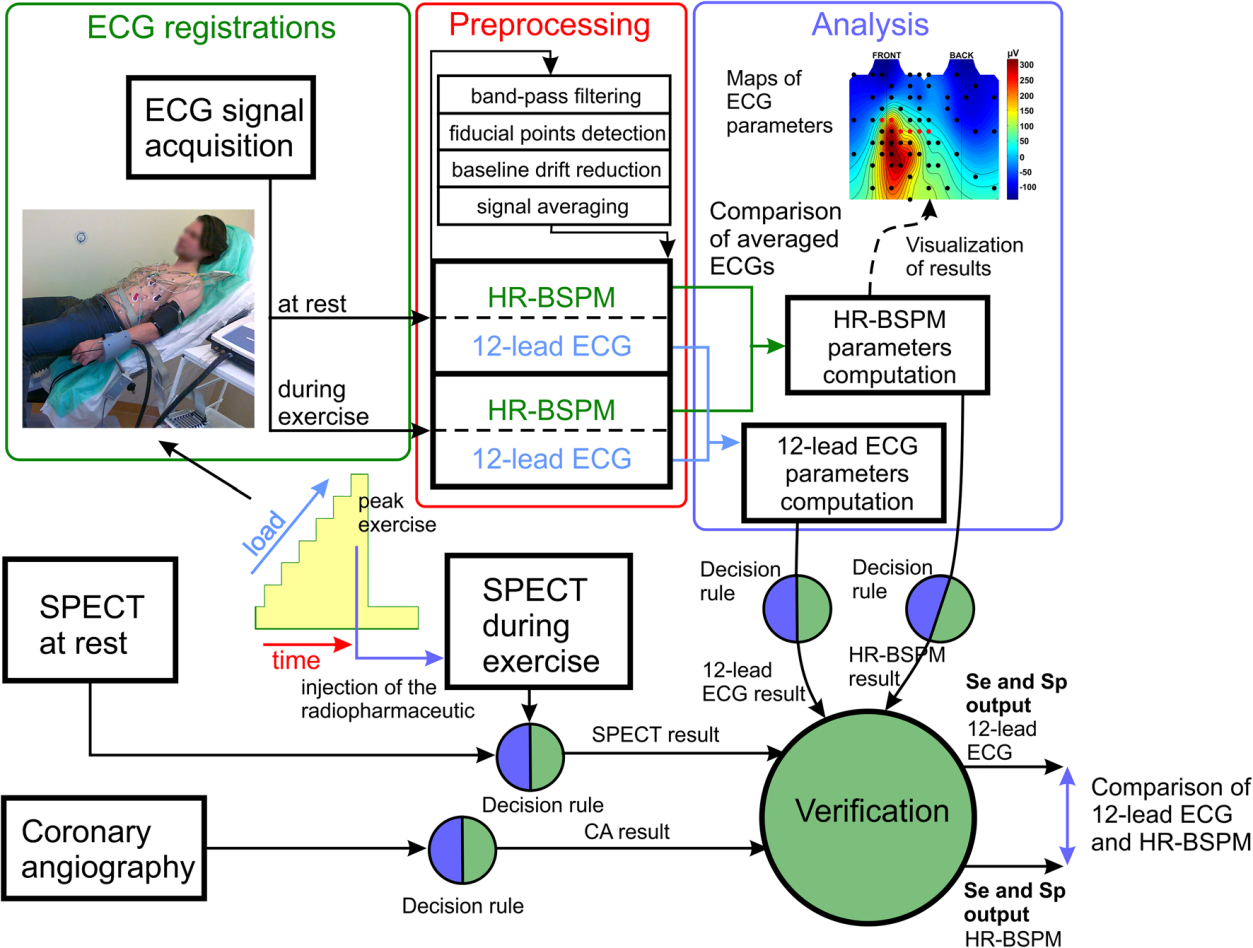
Schematic diagram of a system architecture with the study workflow showing the signal acquisition, preprocessing and analysis as well as graphical presentation of evaluation and decision process.

**Figure 2:**
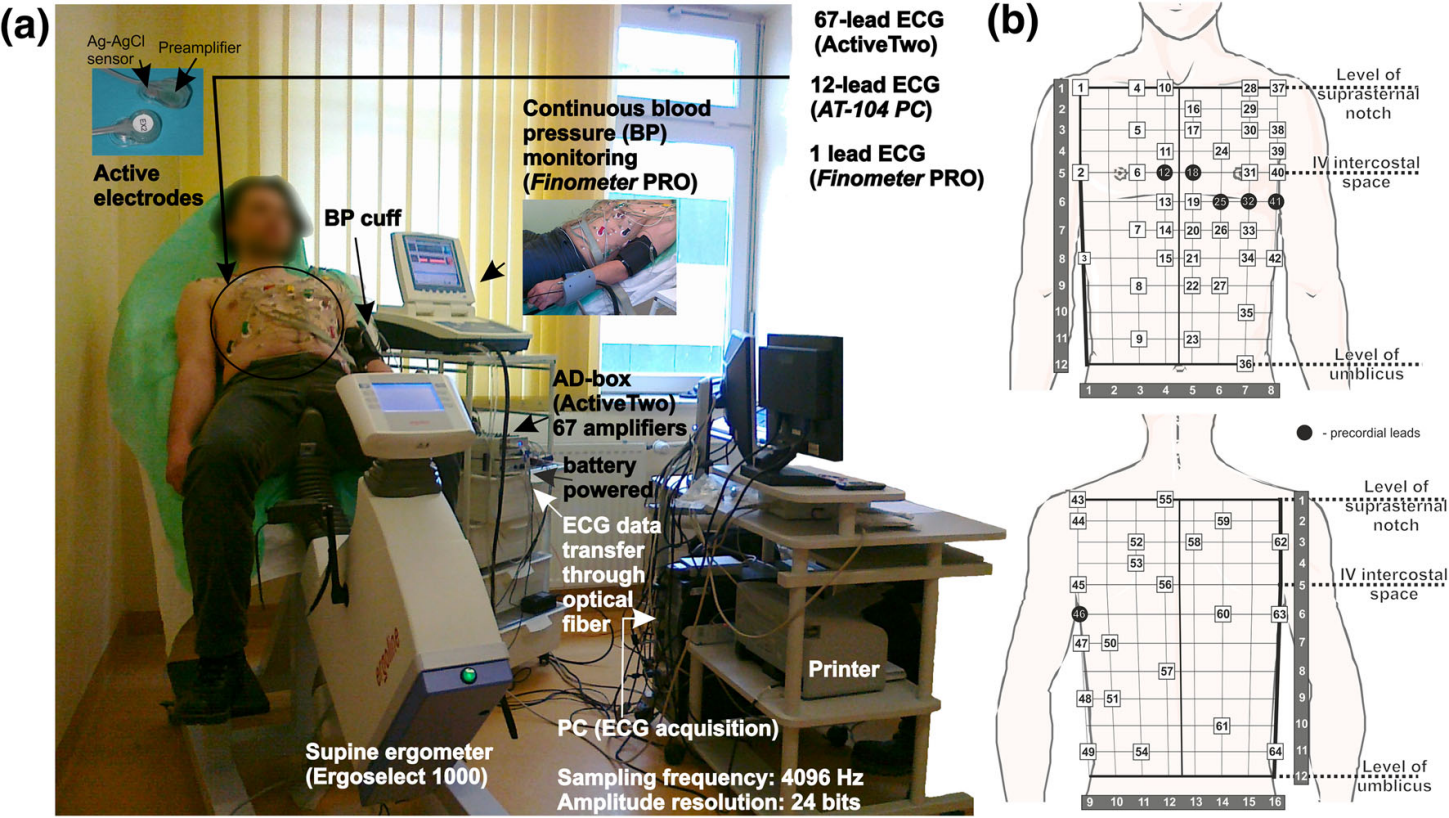
Acquisition system for ischemic heart disease assessment during exercise test (**a**), placement of the ECG electrodes on the thoracic surface (**b**).

The study workflow including data acquisition, as well as preprocessing, analysis, evaluation and decision process are shown in Fig. 1. The exercise test was performed on a supine ergometer (*Ergoselect 1000, Ergoline GmbH*). HR-BSPM data (*AciveTwo, Biosemi BV*), 12-lead ECG (Cardiovit AT-104 PC, *Schiller*) and blood pressure (*Finometer PRO, Finapres Medical Systems*) were simultaneously recorded at rest and during exercise (Fig. 2a).

A 67-channel high-resolution ECG measurement system was used. The system is battery-powered, equipped with active electrodes and fiber optic data transfer to PC, which reduces noise content in recorded data and gives protection for the patient. The 67 active ECG electrodes were placed on the thoracic surface. Specifically, the ECG electrode system developed by Fereniec *et al*.^4^ was used (Fig. 2b), which is an extended version of the Amsterdam leads system.^10^ Limb electrodes were positioned on the shoulders and anterior superior iliac spines to reduce the influence of muscle noise artifacts on recorded signal.

After 10 min recording at rest exercise begun. The workload was increased in stages by 25 W every 2 min, beginning at 50 W. ECG signals were recorded with 4096 Hz sampling frequency and digitized with 24-bit amplitude resolution. The exercise was interrupted when the heart rate reached at least 85% of the maximal predicted value (HR_max_ = 220 − age). The recording of ECG signals was continued at rest for 10 min. Tests were terminated due to chest pain, fatigue, arrhythmias, or marked ST-segment change. All subjects had a diagnostic result from the exercise test, were in sinus rhythm, and were without clinical evidence of bundle branch block, intra-ventricular conduction defects or ventricular hypertrophy. 99mTc-sestamibi was intravenously injected at rest 1 day before the exercise test and on the day of the exercise test, 1 min before cessation of exercise. Within 2 h after radiopharmaceutical injections, scintigraphic imaging was performed using a gamma camera with a 54 × 40 cm^2^ field of view (*Elscint VariCam, Elscint Ltd.*).

Coronary angiography was performed at the Department and Cardiology of the Medical University of Warsaw in accordance with current guidelines for diagnosis and treatment of CAD. Access from the radial artery was preferred. Diagnostic tests were performed on the Philips Allura Xper FD10 cardioangiograph using low iodine and low osmolar contrasts according to standard projections. The images were evaluated by two independent experienced interventional cardiologists.

### Processing of ECG Signals

The ECG signals were band-pass filtered with cut-off frequencies 0.05 and 250 Hz. Baseline wander was reduced using the third-degree polynomial method with reference points computed in each beat as the average amplitude of four samples before the beginning of the QRS complex. ECG signals from each of 67 leads were then averaged in time from up to 2 min measured at rest and from a 10 s interval measured before peak exercise. ECG beats were aligned with the selected “template beat” based on the highest cross-correlation value and accepted for averaging if the coefficient was higher than 0.96. Noisy beats and ventricular extrasystoles were automatically rejected. ECG fiducial points detection was based on analysis of the root-mean-square signal derived from all measured ECG leads. A description of this preprocessing stage is given in Fereniec *et al*.^4^

### Data Analysis

Twenty-one parameters (Table 2) were computed in each ECG lead to describe exercise-induced changes in action-potential distribution on the thoracic surface and to identify those related to IHD.

**Table 2:** ECG parameters used to describe exercise-induced changes in the ECG signal.

No.	Parameter	Description
Amplitude parameters		
1	ΔEA_QRS_	Difference in extreme amplitudes of the QRS complex (µV)
2	ΔST60	Difference in ST segment amplitudes measured 60 ms after J point (µV)
3	ΔEA_T_	Difference in extreme amplitudes of the T wave (µV)
4	Δ*M*_QRS_	Difference in average amplitudes of the QRS complex (µV)
5	Δ*M*_ST_	Difference in average amplitudes of the ST segment (from J point to 80 ms after the J point) (µV)
6	Δ*M*_ST–T_	Difference of in average amplitudes of the ST–T complex (µV)
Time parameters		
7	Δ*t*_QRS_	Difference of QRS complex durations (ms)
8	Δ*t*_ST–T_	Difference of in ST–T complex durations (ms)
ECG signal morphology change		
9	ΔTSI	The change in T-wave shape index of signals *s*_ex_ and *s*_rest_
10–12	*δ*_*s*_	Shape change of signals *s*_ex_ and *s*_rest_ (ms)
13–15	*R*_*s*_	Cross-correlation coefficient between signals *s*_ex_ and *s*_rest_
16–18	RMSD_s_	Root mean squared deviation between signals *s*_ex_ and *s*_rest_ (µV)
19–21	NRMSD_s_	Normalized root mean squared deviation between signals *s*_ex_ and *s*_rest_ (%)

The difference parameters (Δ) refer to the difference between parameter values determined from averaged in time ECG signals recorded at peak exercise (*s*_ex_) and at rest (*s*_rest_). Subscript *s* investigated segments of ECG beat: QRS complex, ST segment or T wave

The amplitude parameters i.e. extreme amplitudes of QRS complex (EA_QRS_) and T wave (EA_T_), the mean amplitudes of QRS complex (*M*_QRS_), ST segment (*M*_ST_), and ST–T complex (*M*_ST–T_), and ST segment amplitude measured 60 ms after J point (*ST60*) were computed. Next, the durations of QRS complex (*t*_QRS_) and ST–T complex (*t*_ST–T_) were determined.

T-wave shape index (*TSI*)^5^–^7^ was computed, defined as the ratio of the area under the T wave and the length of the T-wave curve:

$${\text{TSI}} = \frac{{\mathop \sum \nolimits_{{T_{b} }}^{{T_{e} }} V(t)}}{n \cdot L\left( V \right)},$$

where *V*(*t*) is the amplitude of the ECG signal in time instant *t*, *n* number of samples in T wave, *T*_*b*_ and *T*_*e*_ are respectively the beginning and the end of the T wave. *L*(*V*) is the length of the T wave. *L*(*V*) was determined as the sum of lengths of small sections of T-wave approximated using the Pythagorean theorem.

The exercise-induced changes of aforementioned parameters were assessed by computing the difference of their values determined from ECG signals recorded at peak exercise (*X*_ex_) and at rest (*X*_rest_): Δ*X* = *X*_ex_ − *X*_rest_, where *X* the parameter name: *EA*_QRS_, *EA*_T_, *M*_QRS_*, M*_ST_, *M*_ST–T_, *ST60*, t_QRS_, t_ST–T_, and *TSI*.

**Figure 3:**
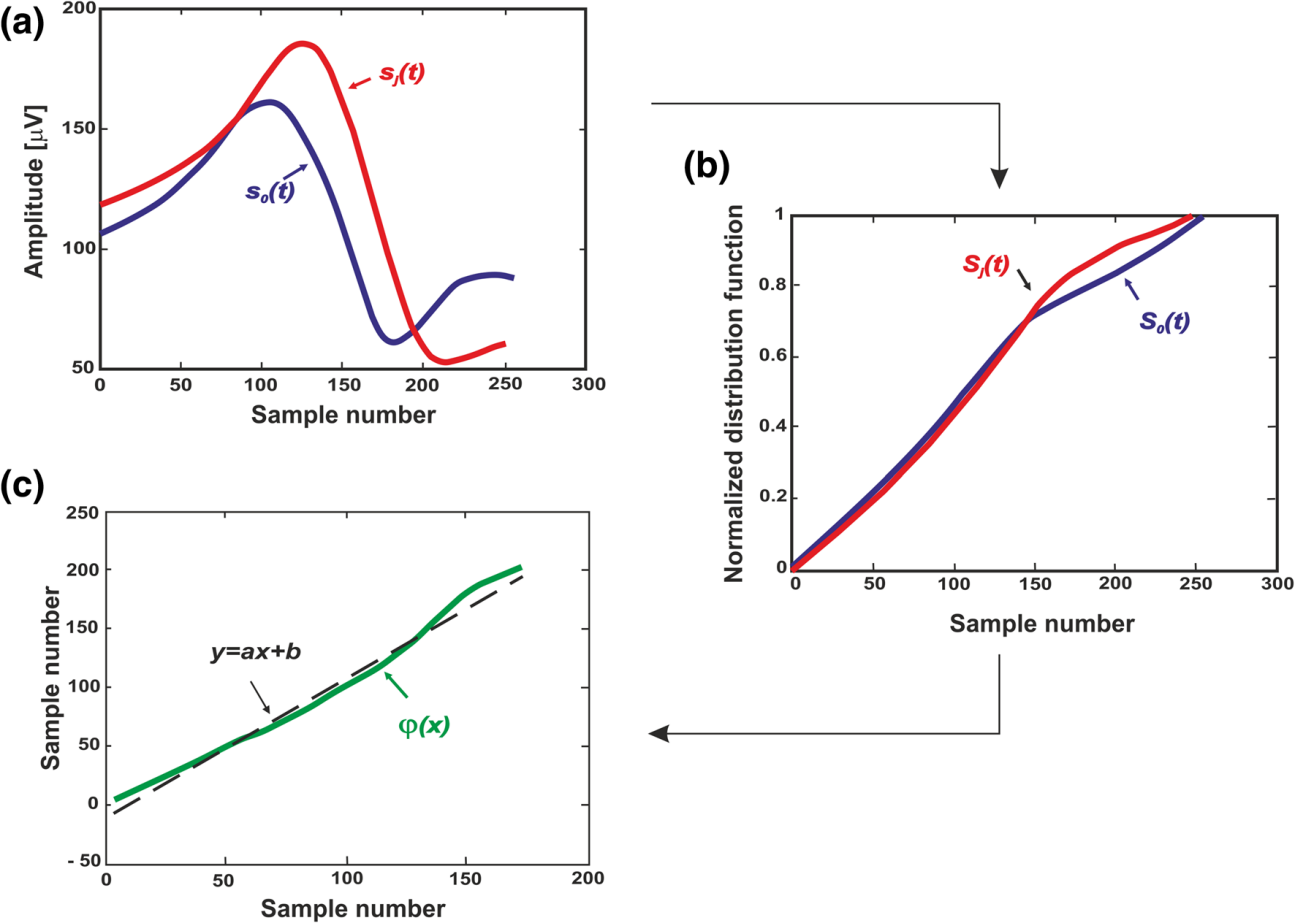
Procedure for calculating the *δ* parameter: (**a**) the two compared signals *s*_*j*_(*t*) and *s*_0_(*t*), (**b**) the normalized integral functions *S*_*j*_(*t*) and *S*_0_(*t*), (**c**) the least mean square line *y*(*t*) fitted on $\varphi$.

The Distribution Function Method (DFM)^31^ was used to precisely evaluate exercise-induced changes in ECG morphology. Let *s*_0_(*t*) be a reference signal (recorded at rest) and *s*_j_(*t*) a signal to compare (recorded at peak exercise). The difference in shape between signals s_0_(t) and s_j_(t) (Fig. 3, a) was characterized by a function $\varphi$ defined by the relation:

$$S_{j} \left( t \right) = S_{0} \left( {\varphi \left( t \right)} \right) {\text{i}}.{\text{e}}.\varphi = S_{0}^{ - 1} \circ S_{j} ,$$

where *S*_j_(*t*) and *S*_0_(*t*) (Fig. 3b) are the normalized integral functions of *s*_j_(*t*) and *s*_0_(*t*) respectively, increasing from zero to one. The shape variation between *s*_j_(*t*) and *s*_0_(*t*) can be quantified by *δ* parameter measuring the distance between the function $\varphi$ and the least mean square line y(t) fitted on $\varphi$^16^ (Fig. 3c):

$$\delta = \sqrt {\frac{1}{n}\mathop \sum \limits_{t = 1}^{t = n} \left( {\varphi \left( t \right) - y\left( t \right)} \right)^{2} } ,$$

where *n* is a number of samples in analyzed window (QRS complex for *δ*_QRS_, ST segment for *δ*_ST_ and T wave in case of *δ*_T_).

The *δ* parameter describes the ‘real’ ECG morphology changes between a pair of two signals omitting the scaling effect i.e. stretching or shrinking of ECG waves either in amplitude or in time. A value of *δ* close to zero means that the shape of signal does not change, *δ* > 0 indicates the appearance of additional components, e.g. the change of the shape of the QRS complex into a rSr’ shape.

The correlation coefficient (*R*) between s_j_ and s_0_ was defined as:

$$R = \frac{{\mathop \sum \nolimits_{t = 1}^{n} s_{0} \left( {\text{t}} \right)s_{j} \left( {\text{t}} \right) - n\overline{{s_{0} }} \overline{{s_{j} }} }}{{\sqrt {\left( {\mathop \sum \nolimits_{t = 1}^{n} s_{0} \left( {\text{t}} \right)^{2} } \right) - n\overline{{s_{0} }}^{2} } \sqrt {\left( {\mathop \sum \nolimits_{t = 1}^{n} s_{j} \left( {\text{t}} \right)^{2} } \right) - n\overline{{s_{j} }}^{2} } }}.$$

where *s*_j_ and *s*_0_ are signals (QRS complex for *R*_*QRS*_, ST segment for *R*_*ST*_, or T wave for *R*_T_) recorded at peak exercise and at rest, $\overline{{s_{0} }}$ and $\overline{{s_{j} }}$ are their means, *n* number of samples in compared signals.

The Root-mean-square deviation (RMSD) between *s*_j_(*t*) and *s*_0_(*t*) was defined by relationship:

$${\text{RMSD}} = \sqrt {\frac{1}{n}\mathop \sum \limits_{t = 1}^{t = n} \left( {s_{0} \left( t \right) - s_{j} \left( t \right)} \right)^{2} } ,$$

where *n* number of samples in analyzed window (QRS complex for RMSD_QRS_, ST segment for RMSD_ST_, or T wave for RMSD_T_). The value of the *RMSD* is influenced by the torso dimension of a given patient. The distance between electrodes and their location in relation to the signal source affects the maximum amplitude of the ECG signal, and thus the RMSD value. To reduce this effect, RMSD was normalized to the range of amplitudes (*s*_min,_*s*_max_) in compared signals (QRS complex − *NRMSD*_*QRS*_, ST segment − *NRMSD*_*ST*_, or T wave − *NRMSD*_*T*_) as follows:

$${\text{NRMSD}} = \frac{\text{RMSD}}{{s_{\hbox{max} } - s_{\hbox{min} } }}.$$

### Statistical Analysis

The effectiveness of ECG parameters in distinguishing groups of patients with and without myocardial ischemia was studied. The Obtained values of ECG descriptors were compared in groups of subjects separated based on SPECT and CA outcomes, i.e. patients with/without IHD detected by SPECT (grSPECT+/grSPECT−), with/without IHD detected by CA (grCA+/grCA−), and with/without IHD detected by both SPECT and CA (grSPECT+CA+/grSPECT−CA−).

Results were presented as mean values of the ECG parameters and the corresponding standard deviations (SD). The assessment of statistical significance of differences between group means was performed using a nonparametric Mann–Whitney test and a *p* value < 0.05 was considered statistically significant. The Matlab “Statistics Toolbox” was used. Effectiveness of the HR-BSPM and classical ECG exercise test in the diagnosis of IHD was evaluated based on the calculated sensitivities and specificities of ECG parameters, defined as:

$${\text{Se}} = \frac{\text{The number of correctly detected subjects with IHD }}{\text{The total number of subjects with IHD}} \cdot 100\% = \frac{\text{TP }}{{{\text{TP}} + {\text{FN}}}} \cdot 100\% ,$$

$${\text{Sp}} = \frac{\text{The number of correctly detected subjects without IHD }}{\text{The total number of subjects without IHD}} \cdot 100\% = \frac{\text{TN}}{{{\text{TN}} + {\text{FP}}}} \cdot 100\% ,$$

where TP—the number of true positive results, TN—the number of true negative results, FN—the number of false negative results, and FP—the number of false positive results.

The Se and Sp of ECG parameters was determined in relation to two reference methods: SPECT and CA separately, and to both methods simultaneously. Incorrect SPECT test result (SPECT+) was defined as the presence of perfusion disorders covering a minimum of 2 out of 17 segments or at least 10% of the myocardium. Coronary artery stenosis above 50% was considered an abnormal CA (CA+).

Receiver Operating Characteristic (ROC) curves were used for comparison of introduced diagnostic methods, which are a cumulative description of the Se and Sp of a given diagnostic parameter for different classifier values. Optimal diagnostic thresholds for ECG parameters were calculated by stepping the threshold value from the minimum value of the parameter observed in ECG maps to its maximum value. For each threshold value, the Se and Sp of the parameter was determined. The point of intersection of Se and Sp curves was chosen as the optimal decision threshold (DT). The two ROC analysis were performed. The first one where fulfillment of the condition “ECG parameter ≥ threshold” was considered as positive result, and second rule with opposite sign (“ECG parameter ≤ threshold”). Finally, a diagnostic threshold rule characterized by the highest values of Se and Sp was chosen for further comparison of the methods. The areas under the ROCs (AUC) were calculated in order to compare the results across the full decision range.

## Results

**Figure 4:**
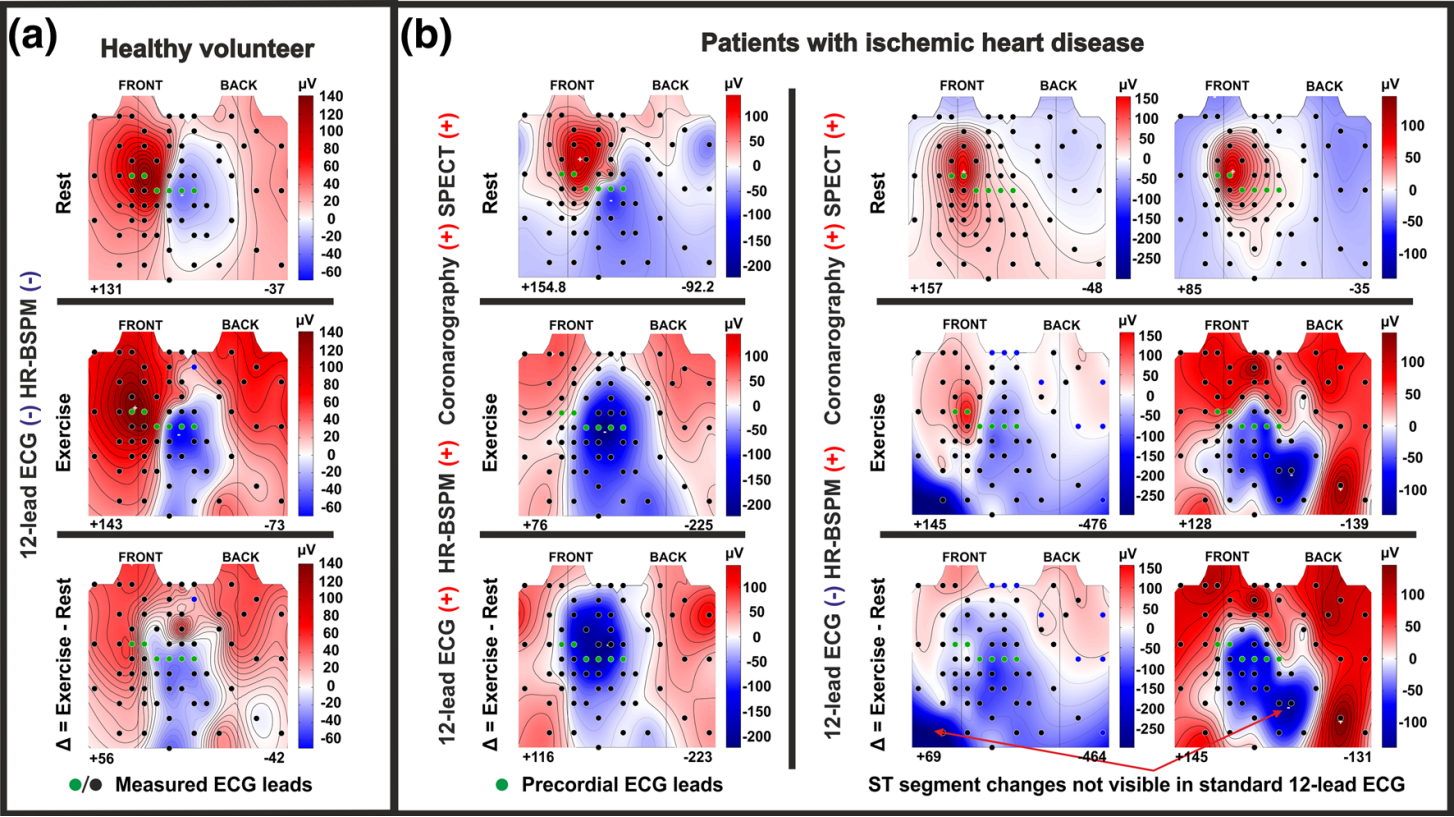
Maps of ST-segment amplitude changes (ST60) for a healthy volunteer (**a**) and three patients with IHD (**b**) recorded at rest and submaximal exercise. Circles on maps indicate the positions of ECG electrodes (those in green color are precordial lead positions). ST-segment elevation is shown in red color, ST depression is highlighted in blue color. The results of the IHD diagnostic tests are shown on the left side of the maps. They are labeled with “+” or “−” indicating respectively positive (detected IHD) and negative (not detected IHD) test result.

The changes induced by exercise were seen for most of the analyzed ECG parameters. The direction of changes i.e. an increase or decrease of parameter value during exercise, depended on: exercise load, the choice of investigated ECG interval and the placement of ECG electrode on the thoracic surface. Examples of ST60 parameter distributions around the torso are shown in Fig. 4. In each column the maps of the ST-segment amplitude for one subject are shown. Starting from the top row, maps recorded at rest, at peak exercise and their difference maps are presented, respectively.

Separate comparisons between groups of subjects were performed for minimal and for maximal values of parameters determined in each map. They refer to the highest observed changes in the ECG signal induced by exercise. For each parameter the most effective method of map assessment was selected with the highest performance in separation of studied groups. Obtained results are shown in Table 3. Those with statistically significant differences (*p* < 0.05) are marked by *.

**Table 3:** Mean changes of ECG parameters ± SD at peak exercise for HC, grSPECT+ and grSPECT−.

Parameter	HC	*p* ^†^	grSPECT+	*p* ^‡^	grSPECT−
QRS complex					
$\Delta {\text{EA}}_{\text{QRS}}^{\hbox{min} }$ (µV)	− 491 ± 208	0.28	− 776 ± 889	0.088	− 511 ± 409
$\Delta M_{\text{QRS}}^{\hbox{max} }$ (µV)	137 ± 60	0.003*	113 ± 90	0.289	108 ± 53
$\delta_{\text{QRS}}^{ \hbox{max} }$ (ms)	2.34 ± 2.45	0.200	3.42 ± 3.76	0.042*	2.15 ± 2.91
$R_{\text{QRS}}^{ \hbox{max} }$	1.00 ± 0.00	0.37	0.97 ± 0.08	0.040*	0.99 ± 0.05
${\text{RMSD}}_{\text{QRS}}^{ \hbox{max} }$ (µV)	281 ± 82	0.16	383 ± 273	0.092	299 ± 164
${\text{NRMSD}}_{\text{QRS}}^{ \hbox{max} }$ (%)	30 ± 7	0.420	31 ± 9	0.24	30 ± 9
$\Delta t_{\text{QRS}}^{ \hbox{min} }$ (ms)	− 8 ± 6	0.4	− 9 ± 8	0.46	− 8 ± 7
ST segment					
Δ*ST60*^min^ (µV)	− 76 ± 41	< 0.001*	− 133 ± 69	0.001*	− 92 ± 54
$\Delta M_{\text{ST}}^{ \hbox{min} }$ (µV)	− 105 ± 52	0.002*	− 145 ± 70	0.016*	− 118 ± 57
$\delta_{\text{ST}}^{ \hbox{max} }$ (ms)	0.19 ± 0.12	0.001*	0.15 ± 0.10	0.50	0.14 ± 0.10
$R_{\text{ST}}^{ \hbox{max} }$	0.97 ± 0.03	0.33	0.97 ± 0.04	0.50	0.97 ± 0.03
${\text{RMSD}}_{\text{ST}}^{ \hbox{max} }$ (µV)	153 ± 62	0.27	164 ± 73	0.026*	138 ± 58
${\text{NRMSD}}_{\text{ST}}^{ \hbox{max} }$ (%)	73 ± 9	< 0.001*	80 ± 8	0.040*	78 ± 7
T wave					
$\Delta EA_{T}^{ \hbox{min} }$ (µV)	− 302 ± 171	0.053	− 365 ± 181	0.025*	− 272 ± 171
ΔTSI^max^ (µV)	50 ± 17	0.007*	41 ± 17	0.34	42 ± 17
$\delta_{T}^{\hbox{max} }$ (ms)	0.53 ± 0.42	< 0.001*	0.87 ± 0.40	0.032*	0.71 ± 0.25
$R_{T}^{ \hbox{max} }$	0.98 ± 0.01	0.042*	0.97 ± 0.02	0.27	0.97 ± 0.02
${\text{RMSD}}_{T}^{\hbox{max} }$ (µV)	257 ± 121	0.13	284 ± 117	0.035*	243 ± 121
${\text{NRMSD}}_{T}^{\hbox{max} }$ (%)	68 ± 9	< 0.001*	58 ± 8	0.006*	62 ± 7
$\Delta t_{T}^{\hbox{min} }$ (ms)	− 108 ± 26	0.002*	− 86 ± 35	0.051	− 99 ± 31
ST–T complex					
$\Delta M_{ST - T}^{ \hbox{min} }$ (µV)	− 147 ± 81	0.043*	− 178 ± 83	0.023*	− 144 ± 72

Superscripts ^min^ or ^max^ refers to the method of computation of mean values: from minima or maxima of map, respectively

*p < 0.05

^†^Compared groups HC and grSPECT+

^‡^Compared groups grSPECT+ and grSPECT−

From the depolarization phase parameters, only *δ*_*QRS*_ and *R*_*QRS*_, which describe the changes in QRS morphology, showed significant differences between the groups of patients with and without detected IHD (*p* values were 0.042 and 0.040, respectively). Their significance levels were lower than for ECG parameters representing the repolarization phase of the heart (Table 3). Therefore, in further analysis we focused on the repolarization period and on those parameters characterized by the lowest *p* values. Results for the depolarization phase were described in detail in Ref. 16.

Myocardial ischemia was associated with changes in the amplitude and morphology of the ECG signal. No statistically significant changes were observed for ECG intervals durations. Ischemic changes were the most pronounced in the ST segment amplitude changes (Δ*ST60*, Δ*M*_*ST*_) and T-wave amplitude changes (Δ*EA*_*T*_), as well as in parameters (*δ*_*T*_, *NRMSD*_*T*_) describing differences in ECG morphology.

**Figure 5:**
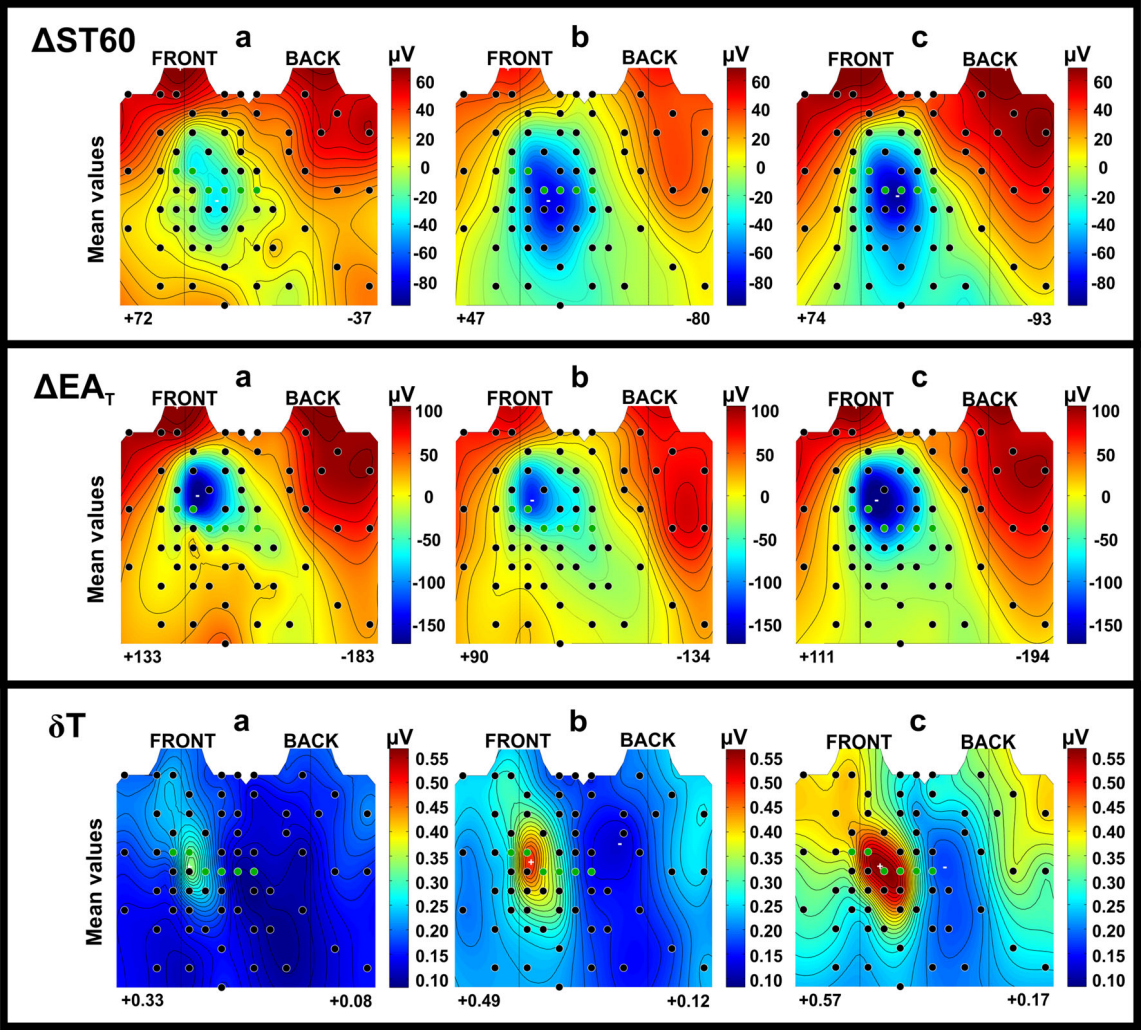
Maps of mean Δ*ST60*, Δ*EA*_*T*_, *δ*_*T*_: (**a**) for HC group, (**b**) for grSPECT- and (**c**) for grSPECT+.

In Fig. 5 the average ΔST60 maps are shown. The decrease of ST segment amplitude during exercise was greatest in the left and in the center part of the chest, mainly in the inferior precordial ECG leads (green circles in Fig. 5). In grSPECT- and grSPECT+, the area of *ST60* reduction expanded significantly to include almost the entire surface of the left side of the chest, as well as expanding on the right-lower part of the chest and left-lower part of the back. The range of *ST60* decrease for patients with diagnosed IHD was larger than for patients without detected ischemia, which were on average − 133 ± 69 and − 92 ± 54 µV (*p* < 0.001, Table 3), respectively. There was also an increase in the SD for the *ST60* means, particularly in the precordial area and left-lower part of the chest. Interindividual differences in *ST60* distributions can be seen clearly in the individual ECG maps presented in Fig. 4. Exercise-induced ST depression was significantly greater in patients with IHD and often appeared in measurement positions outside the precordial leads area.

Exercise-induced changes associated with myocardial ischemia have also been detected in the individual maps of the mean amplitudes of ST segment (Δ*M*_*ST*_), with statistically significant differences observed between grSPECT+ and grSPECT− (*p* = 0.016, Table 3). There were no significant differences related to IHD for *δ*_ST_ (*p* = 0.50) in contrary to RMSD_ST_ (*p* = 0.026) and NRMSD_ST_ (*p* = 0.040).

In the T wave, exercise-induced ischemia was detectable in maps of T-wave amplitude (Δ*EA*_*T*_) and maps of T-wave morphology changes (*δ*_*T*_, *RMSD*_*T*_*, NRMSD*_*T*_). In the precordial leads, at peak exercise, the T-wave amplitude decreases. The area of the amplitude reduction covers mainly the left side of the chest with the maximal decrease under the *V*_2_ lead position (Fig. 5). For IHD patients, the area of ΔEA_T_ changes in a similar way to Δ*ST60*, covering almost the whole surface of the left side of the chest.

The greatest changes in the morphology of the T wave during exercise appeared in *V*_2_ and *V*_3_ lead locations (Fig. 5). However, in the case of IHD patients, those changes were more pronounced and visible across a much larger area on the thoracic surface with the biggest T-wave shape changes often occurred outside the standard ECG leads positions. T-wave morphology changes on the right-upper torso appeared more frequently in grSPECT+ (Fig. 5c) than in grSPECT− (Fig. 5b).

The Se and Sp values in identification of patients with IHD were computed for ECG parameters characterized by the lowest *p* values (Table 3). Obtained results are shown in Table 4. Calculations were made for both the standard 12-lead ECG as well as for the 67-lead ECG layout. Decision thresholds used were determined from ROC curves (see Sect. 1.5). Se’s and Sp’s were computed based on SPECT outcome (grSPECT + and grSPECT−, Table 1).

**Table 4:** Comparison of diagnostic values of selected ECG parameters with reference to SPECT (grSPECT+ vs. grSPECT−).

Parameter	12-lead ECG				HR-BSPM			
	Se	Sp	DT	AUC	Se	Sp	DT	AUC
ST segment								
ΔST60 (µV)	58	59	≤ − 92	0.55	65†	64†	≤ − 99	0.68
Δ*M*_ST_ (µV)	62	62	≤ − 120	0.63	65†	64†	≤ − 128	0.65
NRMSD_ST_ (%)	60†	59†	≥ 1	0.59	56	57	≥ 1	0.61
T-wave								
ΔEA_T_ (µV)	56	57	≤ − 230	0.60	62†	62†	≤ − 270	0.63
*δ*_*T*_ (ms)	60†	62†	≥ 0.70	0.60	60†	62†	≥ 0.75	0.61
NRMSD_T_ (%)	44†	43†	≤ 0	0.43	42	43	≤ 0	0.41
ST–T complex								
Δ*M*_ST–T_ (µV)	54	55	≤ − 127	0.60	60†	59†	≤ − 136	0.60

^†^The highest (12-lead ECG vs. HR-BSPM) obtained values of Se and Sp

The highest values of Se and Sp for separation of the group of patients with and without IHD were in Δ*ST60*, Δ*M*_*ST*_, Δ*EA*_*T*_, and *δ*_*T*_ (Table 4). Those parameters were also characterized by higher AUC. The Se and Sp values computed for HR-BSPM were higher than for the standard 12-lead ECG for all selected parameters (Δ*ST60*, Δ*M*_*ST*_, Δ*EA*_*T*_), except *δ*_*T*_ for which the same values of Se and Sp for both electrode systems were obtained (Table 4).

In the comparison table (Table 4), the method used for Sp calculation with reference to grSPECT- allows assessment of the ability of the test to detect patients with exercise induced ischemia among the patients clinically suspected with CAD. This study represents the clinical situation where the ECG exercise testing is used to check whether a patient should be referred for revascularization procedures. The cardiac diagnostics frequently identifies patients with previously identified heart diseases, e.g. with a history of myocardial infarction. Coexisting cardiac problems affect the ECG signal measured from the thoracic surface and are one of the reasons that the task to evaluate ischemia in this case is hard to achieve.

The results of applying the same selected set of ECG parameters to the task of discrimination between groups of patients with IHD (grSPECT+, Table 1) and healthy controls (HC, Table 1) indicate similarly *δ*_*T*_, *ΔST60* and Δ*EA*_*T*_ as the most effective for an identification of IHD. The obtained Se’s and Sp’s (Table 5, comparison case 1) were higher than in the case of separation of grSPECT+ and grSPECT− (Table 4). For the 67-lead ECG layout these were found to be 73 and 73% (*δ*_*T*_), 71 and 71% (Δ*ST60*), 65 and 65% (Δ*EA*_*T*_), respectively.

**Table 5:** The sensitivity and specificity of parameters ΔEA_*T*_, *δ*_*T*_, and Δ*ST60* in IHD diagnostic.

Comparison case	Compared groups		12-lead ECG			HR-BSPM		
			ΔEA_T_	δ_T_	ΔST60	ΔEA_T_	δ_T_	ΔST60
1	grSPECT+/HC	Se	50	77	67	65	73	71
		Sp	50	76	68	65	73	71
		DT	≤ − 243	≥ 0.53	≤ − 81	≤ − 288	≥ 0.59	≤ − 96
		AUC	0.55	0.82	0.67	0.71	0.80	0.75
2	grCA+/HC	Se	46	77	62.8	58	74	65
		Sp	47	76	64.7	59	73	65
		DT	≤ − 230	≥ − 0.81	≤ − 75	≤ − 229	≥ 0.58	≤ − 95
		AUC	0.49	0.52	0.68	0.60	0.78	0.73
3	grCA+/grCA−	Se	49	53	53	49	56	60
		Sp	48	52	56	48	56	60
		DT	≤ − 221	≥ 0.63	≤ − 86	≤ − 252	≥ 0.72	≤ − 97
		AUC	0.49	0.54	0.56	0.49	0.55	0.61
4	grSPECT+CA+/HC	Se	52	78	67	63	78	74
		Sp	50	76	68	62	76	73
		DT	≤ − 242	≥ 0.54	≤ − 81	≤ − 242	≥ 0.59	≤ − 112
		AUC	0.52	0.83	0.73	0.63	0.83	0.77
5	grSPECT+CA+/grSPECT−CA−	Se	56	59	63	55	59	70
		Sp	56	56	62	56	62	69
		DT	≤ − 223	≥ 0.71	≤ − 86	≤ − 261	≥ 0.75	≤ − 97
		AUC	0.57	0.57	0.56	0.57	0.57	0.69

In this comparison the Sp’s were computed with respect to HC, the group of subjects with no reported history of cardiovascular diseases and a negative result of 12-lead ECG exercise test. This has the potential to evaluate the effectiveness of selected ECG parameters in early screening for ischemic heart disease.

The problem with evaluation of proposed diagnostic methods is that the result of Se and Sp calculation strictly depends on the choice of the reference methods for IHD verification. There is no “gold standard” in this respect. The SPECT method could give false-positive results e.g. related to the absorption of gamma quanta by the tissue located between the heart and the gamma camera or scattering of radiation in the fat tissue.^25^ The CA currently recognized as the standard in the diagnosis of CAD is also not ideal. Proper CA does not exclude the presence of unstable atherosclerotic plaque,^29^ it also does not show the changes in the coronary microcirculation,^18^ and the effect of “stealing” of coronary blood flow in patients with congenital coronary artery fistula.^30^

Table 5 summarizes the results of a different method of testing the diagnostic performance of *δ*_*T*_, Δ*ST60* and Δ*EA*_*T*_. Se’s of 12-lead ECG and HR-BSPM were evaluated with respect to: grSPECT+ (case 1, Table 5; and Table 4), grCA+ (cases 2 and 3, Table 5) and grSPECT+CA+ (cases 4 and 5). The Sp’s were evaluated with respect to: HC (cases 1, 2 and 4, Table 5), grSPECT− (Table 4), grCA− (case 3, Table 5) and grSPECT−CA− (case 5, Table 5).

The effectiveness of tested classification methods in differentiation of patients with and without myocardial ischemia was higher if diagnostic values were verified based on the SPECT result (Table 4, and comparison case 1 in Table 5), than by taking CA as a reference (cases 2 and 3, Table 5). Se’s and Sp’s of ECG parameters for IHD detection were also improved, if the values of Sp were determined in HC group (cases 1, 2, 4, Table 5) than if the Sp were calculated in the group of patients with a negative result of the verification methods (Table 4, and cases 3, 5, Table 5).

**Figure 6:**
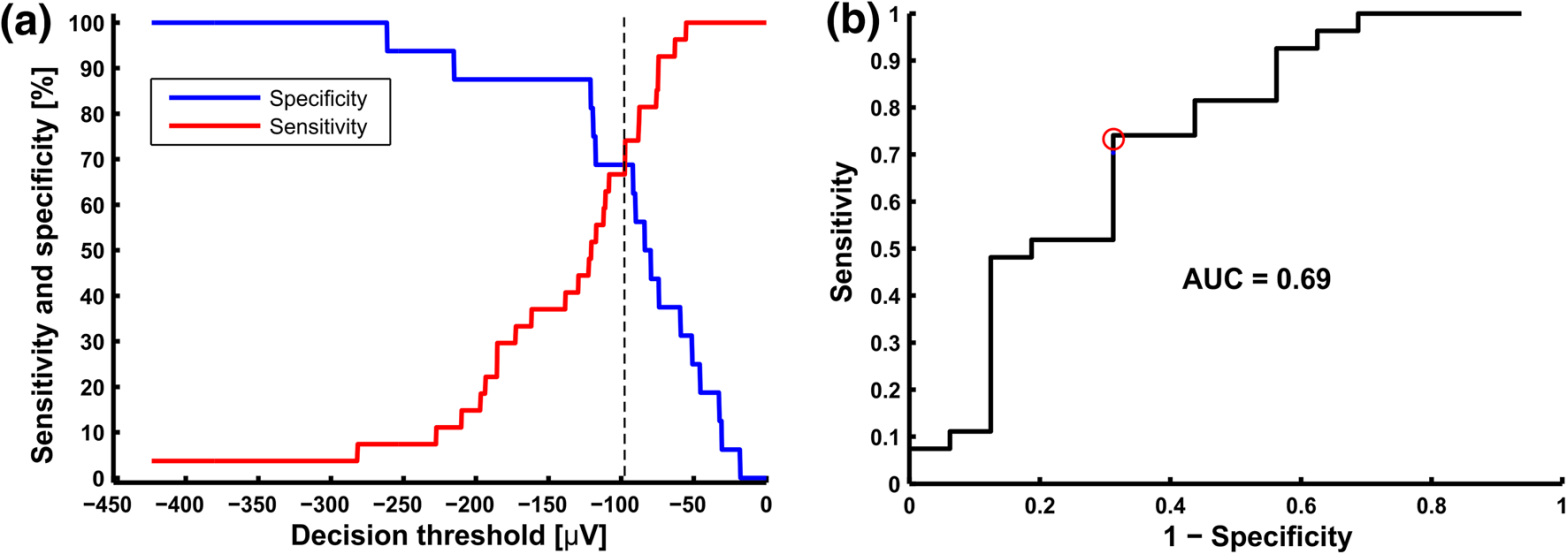
ROC curve analysis for Δ*ST60* calculated from HR-BSPM recordings: (**a**) the modified ROC curve ([Se,Sp] = f(Decision threshold), (**b**) the standard ROC curve (Se =* f*(1 − Sp)). The Se and Sp in a function of decision threshold (≤) were computed in a grSPECT+CA+ and grSPECT−CA−, respectively (Table 5, case 5). Optimal values of Se and Sp at the intersection of curves (**a**) are marked on the panel b with a red circle.

The most effective parameters were the amplitude parameter Δ*ST60* (Fig. 6) and the *δ*_*T*_ parameter describing T-wave morphology changes during exercise. In the group of patients for whom the presence of cardiac ischemia was verified by both SPECT and CA (case 5, Table 5), the Se’s/Sp’s of Δ*ST60* and *δ*_*T*_ parameters for the HR-BSPM were 70/69 and 59/62%, respectively. For the standard 12-lead ECG system Se’s/Sp’s were lower: 63/62 and 59/56%, respectively.

## Discussion

There are few studies that aim to quantitatively evaluate the diagnostic value of body surface potential mapping in the diagnosis of IHD. Available works are mostly descriptive. The authors mainly inform about specific changes in the ECG signal which could be measured from a given electrode locations on the thoracic surface, without verification its diagnostic usefulness. Mentioned BSPM studies were usually performed on small groups of subjects.

Part of the work focuses on the analysis of the effect of coronary artery revascularization procedures. Maynard *et al*.^23^ compared the efficiency of acute coronary syndromes detection using a 80-lead BSPM and classical 12-lead ECG. In the group of 9 patients they analyzed the ST segment amplitude in the J point (ST-0) in 5-s ECG recordings before, during and after percutaneous coronary intervention (PCI). The results of tests indicated BSPM as a method more effective in detecting ECG signal changes, resulting from a sudden closure of the coronary flow. The 53.8% maps of ST-segment amplitude met the criteria for cardiac ischemia compared to 17.9% for the conventional ECG (*p* < 0.001). Specific ischemic changes in the ECG signal, recorded from nonstandard ECG electrode positions, have also been reported in previous studies.^20^,^27^,^36^ Wung *et al*.^36^ analyzed the ST segment amplitude in 68 patients during the occlusion in the branch of the left circumflex artery. The authors used an 18-lead ECG electrode system from which the signals in 192-lead ECG electrode configuration were reconstructed. The biggest changes (elevation or depression) in ST segment were detected outside of the standard ECG electrode positions, which was also confirmed by the results of Kornreich *et al*.^20^

Montague *et al*.^27^ studied the effect of coronary artery revascularization on a group of 24 patients with CAD. They compared the integral maps of QRS, ST and T wave before and after PCI. Successful PCI treatment did not cause significant changes in the depolarization and early repolarization. The biggest changes were observed in the range of the T-wave. Improvement of myocardial perfusion was associated with decreased T-wave amplitude averages. This effect was maintained 24 h after PCI. We observed the opposite effect of ischemia during exercise testing, i.e. a significantly greater reduction in T-wave amplitude in patients with diagnosed CAD as compared to patients without ischemia, as well as for HC group (Fig. 5). The differences likely arise from the fact that exercise-induced ischemia mostly covers subendocardium, while cardiac ischemia induced by sudden occlusion of the coronary arteries have transmural nature.^22^ The effect of changing the location of ischemic area in the heart is the change in the direction of injury current, which determines the polarity of the ECG signal amplitudes recorded from the body surface.

Montague *et al*.^26^ described the impact of physical activity on the maps of QRS and ST amplitude in the group of 14 patients with IHD and 8 healthy controls. They compared the integral maps of QRS complex and ST segment before and immediately after the ECG exercise test. In healthy controls, physical activity was associated with a small reduction in the ST segment, mainly in the precordial area, returning to the resting value 5 min after the end of exercise. In patients with CAD, greater depressions in the ST segment were observed. Positive amplitudes of ST segment observed at rest become negative after the peak exercise. These changes were observed not only in the precordium, but also in the whole lower part of the torso. However, Montague *et al*.^26^ did not detect significant differences in the mean amplitudes of QRS complex which was also pointed out in our previous study concerning the effect of exercise on QRS complex morphology.^16^

In our study, similar changes in the ST segment to those observed by Montague *et al*.^26^ (Fig. 5, Table 3) were observed. The changes in the ECG signal amplitudes detected at the top of exercise in HC were a little deeper in grSPECT- and the deepest in grSPECT+. Moreover, in patients with detected cardiac ischemia, ST segment amplitude reduction covered a wider area of the thoracic surface. This confirms the hypothesis made by a Montague *et al*.^26^ that ischemic changes are a kind of continuation of the physiological changes in response to exercise and have nature of temporal changes at the cellular level.

Hanninen *et al*.^9^ examined the diagnostic efficiency of Δ*ST60* in body surface potential mapping during exercise test. The analysis included 123 recordings of 123-lead ECG maps for 45 patients and 25 healthy controls. The authors evaluated the Se and Sp of the BSPM in the detection of significantly narrowed coronary arteries. For Δ*ST60* parameter and determined optimal positions of ECG electrodes, the method Se was 84% and Sp was equal to 96%. In our study, the Se and Sp of BSPM method in patients with positive CA and a group of HC were much lower: respectively 65 and 65% (Table 5, case 2). The difference in the results may be associated with a much larger study group used in case of our study, as well as that analysis of ST segment depression was performed in a whole map and not in one optimal ECG lead system.^9^ In addition, they chose for calculations the segment of the ECG signal recorded immediately after exercise. We decided to analyze ECG signals at peak exercise, because in this phase of the exercise test there are the biggest changes in the ST segment amplitude. In addition, the sudden changes in heart rate that occur immediately after end of exercise, can significantly change the morphology of averaged in time ECG signal.^14^

In this study, HR-BSPM outperforms 12-lead ECG in detection of IHD. The higher diagnostic accuracy was obtained by application of simple rules to detect IHD patients: with one decision threshold for a given parameter, for all ECG leads and for all patients. The concept is not so much different in comparison to 12-lead ECG. The main difference is that there are much more ECG leads available that could capture ischemic lesions invisible in standard ECG electrode locations. The HR-BSPM recording during exercise could have much more to offer. For example, the diagnostic value of a multi-parameter analysis or the use of spatial patterns in the maps to non-invasively locate ischemic areas in the heart may be the subject of future studies.

In order to convince medical staff to use HR-BSPM in daily clinical practice, several issues should be noted. The positioning of a large number of ECG electrodes could takes longer time than in case of 12-lead ECG and need additional training. In our case the mean time of electrode positioning was around 20 min. The design of the acquisition system required that each of electrodes need to be placed separately. However, there are BSPM systems available which use e.g. electrode strips (Procardio 8, Slovakia^12^) or electrode vest like CardioInsight from Medtronic,^2^ which drastically reduces and simplifies the procedure of preparation for measurement. In both systems the problem of electrode placement errors can be omitted because the data from CT can be used to know exact locations of ECG electrodes on the thoracic surface with reference to heart position.

Looking at ECG maps is different than to look at standard ECG during exercise. Interpretation of potential distributions in BSPM needs training, but colorful maps of ST segment depression show the ST60 values in all leads in one time, what could be easier to follow in comparison to look at all the trends in case of standard 12-lead ECG. Furthermore, the 12-lead ECG can be easy extracted form BSPM and presented on request.

## Conclusions

The obtained results showed usefulness of HR-BSPM measurements during exercise, especially in the case of evaluation of ischemia in the group of patients with other coexisting cardiac pathologies. Noninvasive HR-BSPM could be used in screening for IHD, for selection of patients who require more accurate, but more expensive or more invasive diagnostic imaging. HR-BSPM resulted in higher sensitivities and specificities compared to standard 12-lead exercise test. The most effective parameters in the diagnosis of IHD were: amplitude parameter Δ*ST60*, the *δ*_*T*_ parameter showing T-wave morphology changes during exercise, and Δ*EA*_*T*_ characterizing T-wave extremal amplitude changes. Higher sensitivities and specificities obtained using HR-BSPM were associated with a number of measuring points. In this study, the deeper ST amplitude changes were observed for IHD patients than for healthy controls and were often visible in ECG electrodes located outside the standard ECG leads positions. This motivates further research into optimization of the number and position of ECG leads in exercise studies of myocardial ischemia.
